# Prediction of Falls and/or Near Falls in People with Mild Parkinson’s Disease

**DOI:** 10.1371/journal.pone.0117018

**Published:** 2015-01-30

**Authors:** Beata Lindholm, Peter Hagell, Oskar Hansson, Maria H. Nilsson

**Affiliations:** 1 Department of Neurology, Skåne University Hospital, Malmö, Sweden; 2 The PRO-CARE Group, School of Health and Society, Kristianstad University, Kristianstad, Sweden; 3 Department of Clinical Sciences, Lund University, Malmö, Sweden; 4 Memory Clinic, Skåne University Hospital, Malmö, Sweden; 5 Department of Health Sciences, Lund University, Lund, Sweden; Oslo University Hospital, NORWAY

## Abstract

**Objective:**

To determine factors associated with future falls and/or near falls in people with mild PD.

**Methods:**

The study included 141 participants with PD. Mean (SD) age and PD-duration were 68 (9.7) and 4 years (3.9), respectively. Their median (q1–q3) UPDRS III score was 13 (8-18). Those >80 years of age, requiring support in standing or unable to understand instructions were excluded. Self-administered questionnaires targeted freezing of gait, turning hesitations, walking difficulties in daily life, fatigue, fear of falling, independence in activities of daily living, dyskinesia, demographics, falls/near falls history, balance problems while dual tasking and pain. Clinical assessments addressed functional balance performance, retropulsion, comfortable gait speed, motor symptoms and cognition. All falls and near falls were subsequently registered in a diary during a six-month period. Risk factors for prospective falls and/or near falls were determined using logistic regression.

**Results:**

Sixty-three participants (45%) experienced ≥1 fall and/or near fall. Three factors were independent predictors of falls and/or near falls: fear of falling (OR = 1.032, p<0.001) history of near falls (OR = 3.475, p = 0.009) and retropulsion (OR = 2.813, p = 0.035). The strongest contributing factor was fear of falling, followed by a history of near falls and retropulsion.

**Conclusions:**

Fear of falling seems to be an important issue to address already in mild PD as well as asking about prior near falls.

## Introduction

Postural instability is one of the cardinal signs of Parkinson’s disease (PD). People with PD are particularly unstable backwards [1–3] which commonly is assessed by using the pull test. Several versions of the pull test exist, of which the Nutt Retropulsion Test (NRT) with an unexpected shoulder pull (1 trial) has been preferred [4]. Besides being able to counteract an externally applied perturbation, it is imperative to maintain balance while performing voluntary and self-generated movements in daily life [5]. That is, functional balance performance is also of importance.

In the postural instability and gait difficulty (PIGD) subtype of PD, walking difficulties and balance demanding activities may be affected already early on [6,7]. Gait and balance problems in PD relate to both motor and non-motor features such as cognitive dysfunction [8]. These problems are often aggravated while performing dual tasks [8], and walking difficulties have been shown to be the strongest associated factor to fear of falling (FOF) in people with PD [9,10].

Falls are one of the most disabling features of PD and occur in 35–90% of patients, among whom 18–65% experience recurrent falls [11]. It is also common for people with PD to experience so called near falls [9,10,12–16], which occur also among those who do not fall (60–62%) [12,13]. Previous studies have identified several risk factors for future falls in PD, such as freezing of gait, balance and mobility problems as well as cognitive impairments and a history of falls (e.g.[17–19]). To the best of our knowledge only one previous PD study included a history of near falls (during the past 12 months) as an independent variable when investigating risk factors for future falls [20]. Falls were however not registered prospectively as recommended [21] but registered based on recall at a three-month follow-up. Although the study by Ashburn et al. did not identify prior near falls as a risk factor [20], it has been suggested that near falls may in fact be a precursor of an increased risk for future falls [22,23]. Near falls may therefore be of specific importance in mild PD. Early detection of those at risk may facilitate preventive means. The objective of this study was therefore to determine factors associated with future falls and/or near falls in mild PD.

## Methods

### Ethics statement

The Regional Ethical Review Board in Lund, Sweden approved the study (Dnr 2011/768). All participants gave written informed consent.

### Participants

All people diagnosed with PD receiving care at a south Swedish university hospital during 2007–2012 were considered eligible for inclusion (n = 349). Exclusion criteria were age above 80 years old (n = 116), inability to stand without support (n = 22), unable to understand instructions (n = 14) or having severe comorbidity (n = 11). Of the remaining 186 potential participants, 40 (16 women) declined participation. Those who declined did not differ significantly (P≥0.061, Mann—Whitney U test) from the 146 participants with respect to age and PD duration.

### Procedure and Instruments

Anti-parkinsonian medications were recorded from medical records. All participants were assessed during an outpatient visit, which was scheduled at a time of day when the participant usually reported to feel at best.

First, the participants completed self-administered questionnaires targeting freezing of gait, turning hesitations [24–26], walking difficulties in daily life [27], fatigue [28,29], FOF (conceptualized as fall-related self-efficacy) [30,31], and independence in activities of daily living [32,33]. Further details are provided in Table 1.

**Table 1:** Descriptions of included self-administered questionnaires and clinical assessments.^1^

	Score range	Dichotomized	References
Cognition (MMSE)	0–30 ^2^		37
Comfortable gait speed m/s (10MWT)	≥ 0 m/s		34
Dyskinesia (item 32, UPDRS IV)	0 (none)–4 (76%–100% a day)	No (0), yes (1–4)	36
Fatigue (FACIT-F)	0–52 ^2^		28, 29
Fear of falling (FES [26])	0–130 ^2^		30, 31
Freezing of gait (item 3, FOGQsa)	0 (never)–4 (always—whenever walking)	No (0), yes (1–4)	24–26
Functional balance (BBS)	0–56 ^2^		5, 34
Motor symptoms (UPDRS III)	0–108 ^3^		36
Need help from others in daily activities (PADLS)	1 (no difficulties with day-to-day activities)—5 (extremedifficulties with day-to-day activities)	No (1–2), yes (3–5)	32, 33
Retropulsion (NRT)	0 (normal, may take 2 steps to recover)—3 (spontaneoustendency to fall or unable to stand unaided, test not executable)	No (0), yes (1–3)	35
Severity of disease (H&Y)	I-V ^3^		38
Turning hesitations (item 6, FOGQsa)	0 (never)—4 (more than 30 seconds)	No (0), yes (1–4)	24–26
Walking difficulties (Walk-12G)	0–42 ^3^		27

^1^ Additional dichotomous questions that were included targeted: history of falls, history of near falls, balance problems while dual tasking and pain.

^2^higher = better

^3^higher = worse

BBS, Berg Balance Scale; FACIT-F, the Functional Assessment of Chronic Illness Therapy—Fatigue; FES(S), Falls Efficacy Scale (Swedish version); FOGQsa, Freezing of Gait Questionnaire, self-administered version; H&Y, Hoehn & Yahr stage; MMSE, Mini Mental State Examination; NRT, Nutt Retropulsion Test; PADLS, the Parkinson’s disease Activities of Daily Living Scale; UPDRS III, part III (motor score) of the Unified Parkinson’s Disease Rating Scale; UPDRS IV, part IV (complications of therapy) UPDRS IV, part IV (complications of therapy) was self-administered; 10MWT, 10-Meter Walk Test; m/s, meters per second; Walk-12G, 12-item generic walking scale.

All participants then self-rated their present motor status as “good/on”, “on with dyskinesias”, or “bad/off”. This was followed by clinical assessments (Table 1) administered by the same physical therapist (BL). These were performed in the following order and targeted: functional balance performance (Berg balance scale, BBS) [5,34]; retropulsion (Nutt retropulsion test, NRT) [4,35]; comfortable gait speed (10-Meter Walk-test, 10MWT) [34]; motor symptoms (Unified Parkinson’s Disease Rating Scale, UPDRS part III) [34,36] and cognition (Mini-Mental State Examination, MMSE) [37]. For descriptive purposes, severity of disease was assessed according to Hoehn & Yahr stage (H&Y) [38].

Additional self-administered questions were then administered. These targeted demographic data (age, sex, disease duration) and the presence or absence of dyskinesia [36] (Table 1). In addition, dichotomous questions (Yes/No) targeted history of falls during the past six months (*In the last six months*, *have you fallen in such a way that your body hit the ground?*), history of near falls (*Are you ever close to falling*, *but you manage to grab on to something/someone at the last minute so that your body does not hit the ground?*), balance problems while dual-tasking (*Do you experience balance problems while standing or walking when doing more than one thing at a time*, *e.g. carrying a tray while walking?*) and pain (*Do you presently suffer from pain?*).

### Prospective assessment of falls and near falls

By using a diary, participants were instructed to register all consecutive falls and near falls for six months. At the outpatient visit, the definitions of a fall and a near fall were thoroughly described to all participants. Falls were described and defined as “*an unexpected event in which the participants come to rest on the ground*, *floor*, *or lower level*” [21]. Near falls were described and defined as”*a fall initiated but arrested by support from the wall*, *railing*, *other person etc*.” [39].

In the diary, questions (Yes/No) clarified whether the incident was a fall or a near fall. The question in relation to a fall was phrased as follows: “*Did you fall in such a way that your body hit the ground?*” The corresponding question about a near fall incident was phrased: “*Were you close to falling*, *but managed to brace yourself at the last moment (e.g. grabbed on to someone*, *to an object or the wall?*”

All participants were telephoned monthly to ensure that registrations were completed according to instructions. During the last telephone call, they were requested to return the diary in a pre-stamped envelope.

### Statistical analysis

Data were checked regarding underlying assumptions and described accordingly using IBM SPSS version 21. Normally distributed interval/ratio level variables were described using means and SDs. In other cases, medians (q1–q3) were used. Categorical variables were described using n (%). The alpha level of significance was set at 0.05 (2-tailed). Anti-parkinsonian medications were expressed as daily levodopa equivalent (LDE) doses (mg/day) [40].

Logistic regression analysis was performed in order to establish risk factors for prospective falls and/or near falls (dependent variable). Initially, simple logistic regression analysis was used for factors (independent variables) that were considered potentially important for falls and/or near falls. Variables that were significant at the alpha level of 0.2 were subsequently included as independent variables in a multiple logistic regression analysis in order to identify those independently associated with prospective falls and/or near falls. The <0.2 P-value threshold was chosen in order to avoid leaving a confounding variable out. Both forward and backward methods were used (Wald test). The final model was controlled for age and gender.

In order to facilitate comparisons with prior studies, we also explored factors associated with prospective falls only (i.e. non-fallers and near falls only vs. fallers). The statistical procedure was otherwise identical to the main analysis (see above).

## Results

Five participants did not complete the prospective 6-month follow-up of falls and/or near falls due to e.g. developing severe comorbidities. The final sample (n = 141; 97%) had a mean (SD; min-max) age and PD duration of 68 (9.7; 35–80) and 4 (3.9; 0.1–17) years, respectively. Their median (q1–q3) UPDRS III score was 13 (8–18). Further details are provided in Table 2.

**Table 2:** Sample characteristics (n = 141).

Age (years), mean (SD)	68 (9.7)
Female gender, n (%)	65 (46)
PD-duration (years), median (q1–q3)	2 (1–6)
Cognition (MMSE), median (q1–q3) ^1^	28 (26–29)
Motor symptoms (UPDRS III), median (q1–q3)	13 (8–18)
Dyskinesia (UPDRS IV, item 32), n (%) ^2, a^	46 (33)
Severity of disease (H&Y), median (q1–q3)	2 (2–3)
Daily total levodopa equivalent (LDE) dose (mg), median (q1–q3) ^b^	400 (286–600)
Dopamine agonist use, n (%)	55 (39)
Functional balance performance (BBS), median (q1–q3)	53 (48–55)
Comfortable gait speed (10MWT, m/s), mean (SD)	1.14 (0.28)
Fatigue (FACIT-F), median (q1–q3)	39 (29.5–44)
Pain, n (%)	40 (28)
Walking difficulties (Walk-12G), median (q1–q3)	8 (4–19.5)
Fear of falling (FES[S]), median (q1–q3) ^3^	118 (84–129)
History of falls past 6 months, n (%)	34 (24)
History of near falls, n (%)	50 (35)
Balance problems while dual-tasking, n (%) ^c^	68 (48)
Need help from others in daily activities (PADLS), n (%) ^1, d^	13 (9)
Freezing of gait (item 3, FOGQsa), n (%) ^1, e^	58 (41)
Turning hesitations (item 6, FOGQsa) n (%) ^1, f^	49 (35)
Retropulsion (NRT), n (%) ^g^	35 (25)

^1^One missing value

^2^Two missing values

^3^Four missing values

^a^Item 32 of the UPDRS part IV. Those scoring ≥1 were categorized as having dyskinesias.

^b^Derived according to Tomlinson et al. (2010).

^c^Investigated with dichotomous question (Yes/No) “*Do you experience balance problems while standing or walking when doing more than one thing at a time*, *e.g. carrying a tray while walking?*”

^d^Those scoring >2 on the PADLS were categorized as needing help from others in daily activities.

^e^Item 3 (“freezing”) of the FOGQsa. Those scoring ≥1 were categorized as freezers.

^f^Item 6 (“turning hesitations”) of the FOGQsa. Those scoring ≥1 were categorized as having turning hesitations.

^g^Scores ≥1 on the NRT were categorized as having retropulsion

BBS, Berg Balance Scale; FACIT-F, the Functional Assessment of Chronic Illness Therapy—Fatigue; FES(S), Falls Efficacy Scale, (Swedish version); FOGQsa, Freezing of Gait Questionnaire, self-administered version; H&Y, Hoehn & Yahr stage; MMSE, Mini Mental State Examination; NRT, Nutt Retropulsion Test; PADLS, the Parkinson’s disease Activities of Daily Living Scale; PD, Parkinson’s disease; q1–q3, 1^st^-3^rd^ quartile; SD, standard deviation; UPDRS III, part III (motor score) of the Unified Parkinson’s Disease Rating Scale; UPDRS IV, part IV (complications of therapy), item 32; 10MWT, 10-Meter Walk Test; m/s, meters per second; Walk-12G, 12-item generic walking scale.

At the time of assessments, 123 out of the 141 participants (87%) rated their motor status as”on”, whereas 12 (9%) rated it as “on with dyskinesias”, and 6 (4%) rated it as”off”.

### Prospective falls and/or near falls

During the 6-month follow-up, 63 out of 141 (45%) participants experienced at least one fall and/or near fall. Forty-five out of 141 participants (32%) reported falls, of whom 26 (58%) reported more than one fall (i.e. recurrent falls); on average they reported 5 falls (min-max, 2–12). In total, 44 participants (31%) reported near falls. Eighteen of those reported only near falls whereas 26 also reported falls. Twenty-six out of the 44 (59%) reported more than one near fall; they reported on average 11 near fall incidences (min-max, 2–45). The total number of all reported incidences was 452 (n = 63); 158 (35%) of those were falls whereas 294 (65%) were near falls.

### Predictors of prospective falls and near falls

Simple logistic regression analyses identified 16 independent variables associated with prospective falls/near falls at P<0.2 (Table 3). Table 4 summarizes the results when entering these independent variables into forward and backward logistic regression analyses controlling for age and gender. Three significant independent predictors for prospective falls/near falls were identified: FOF (FES(S)), history of near falls, and retropulsion (NRT). Results were identical for both forward and backward procedures.

**Table 3:** Descriptive statistics and results from simple logistic regression analyses for potential predictors of future falls and/or near falls.

Independent variables^1^	No falls or near fallsn = 78	Falls and/or near fallsn = 63	Simple logistic regression analyses	
			OR (95% CI)	P value
Age (years) mean (SD)	66 (10.4)	70 (8.3)	–	–
Female gender, n (%)	30 (38)	35 (56)	–	–
PD-duration (years), median (q1–q3)	2 (5–5)	4 (1–8)	1.12 (1.03–1.22)	0.011
Cognition (MMSE), median (q1–q3)	28 (26–29) ^2^	27 (26–29)	1.20 (1.01–1.42)	0.034
Motor symptoms (UPDRS III), median (q1–q3)	11.5 (7–15)	18 (10–23)	1.13 (1.07–1.2)	<0.001
Dyskinesia (UPDRS IV item 32), n (%) ^a^	22 (29) ^3^	24 (38)	1.51 (0.74–3.07)	0.255
Balance (BBS), median (q1–q3)	55 (52–56)	50 (42–54)	1.20 (1.11–1.30)	<0.001
Comfortable gait speed (10MWT) (m/s), mean (SD)	1.24 (0.22)	1.02 (0.31)	21.92 (5.3–90.44)	<0.001
Fatigue (FACIT-F), median (q1–q3)	41.5 (36–47)	31.5 (23–41)	1.11(1.06–1.16)	<0.001
Pain, n (%)	15 (19)	25 (40)	2.76 (1.30–5.89)	0.008
Walking difficulties (Walk-12G), median (q1–q3)	6 (2–10)	17 (9–25)	1.12 (1.07–1,17)	<0.001
Fear of falling (FES[S]), median (q1–q3)	127 (117–130) ^3^	88 (60–122) ^3^	1.03 (1.02–1.05)	<0.001
History of falls past 6 months, n (%)	9 (11.5)	25 (40)	5.04 (2.14–11.90)	<0.001
History of near falls, n (%)	13 (17)	37 (59)	7.12 (3.27–15.50)	<0.001
Balance problems while dual-tasking, n (%)	26 (33)	42 (67)	4.00 (1.92–8.09)	<0.001
Need help from others in daily activities (PADLS), n (%) ^b^	1 (1.3) ^2^	12 (19)	17.88 (2.55–141.80)	0.006
Freezing of gait (item 3, FOGQsa), n (%) ^c^	19 (25) ^2^	39 (62)	4.96 (2.40–10.25)	<0.001
Turning hesitations (item 6, FOGQsa), n (%) ^d^	16 (21) ^2^	33 (52)	4.19 (2.00–8.79)	<0.001
Retropulsion (NRT), n (%) ^e^	11 (14)	24 (38)	3.75 (1.66–8.47)	0.001

^1^ For the regression analysis, scores were adjusted to be in the same direction: higher scores = more problems.

^2^ One missing value

^3^ Two missing values

^a^ Item 32 of the UPDRS part IV. Those scoring ≥1 were categorized as having dyskinesias.

^b^ Those scoring >2 on the PADLS were categorized as needing help from others in daily activities.

^c^ Item 3 (“freezing”) of the FOGQsa. Those scoring ≥1 were categorized as freezers.

^d^ Item 6 (“turning hesitations”) of the FOGQsa. Those scoring ≥1 were categorized as having turning hesitations.

^e^ Scores ≥1 on the NRT were categorized as having retropulsion

Wald test

BBS, Berg Balance Scale; FACIT-F, the Functional Assessment of Chronic Illness Therapy—Fatigue; FES(S), Falls Efficacy Scale, (Swedish version); FOGQsa, Freezing of Gait Questionnaire, self-administered version; MMSE, Mini Mental State Examination; NRT, Nutt Retropulsion Test; PADLS, the Parkinson’s disease Activities of Daily Living Scale; q1–q3, 1^st^-3^rd^ quartile; SD, standard deviation; UPDRS III, part III (motor score) of the Unified Parkinson’s disease Rating Scale; UPDRS IV, part IV (complications of therapy), item 32; 10MWT, 10-Meter Walk Test; m/s, meters per second; Walk-12G, 12-item generic walking scale.

**Table 4:** Multiple logistic regression model: prediction of future falls and/or near falls (n = 135)^1^.

**Independent variables** ^2^	Wald	P-value	OR (95% CI)
Age (years)	0.485	0.486	1.017 (0.969–1.067)
Female gender	1.735	0.188	1.792 (0.752–4.267)
Fear of falling (FES[S])^3^	14.254	<0.001	1.032 (1.015–1.049)
History of near falls	6.750	0.009	3.475 (1.358–8.893)
Retropulsion (NRT)	4.428	0.035	2.813 (1.073–7.373)

^1^Forward/backward method (Wald); Nagelkerke pseudo R-square: 0.450; Hosmer and Lemeshow test: P = 0.438. The model was controlled for age and gender (italics in the table).

^2^Independent variables initially entered in the analysis were: age (years), gender, PD duration (years), cognition (MMSE), motor symptoms (UPDRS part III), functional balance (BBS), 10MWT, (comfortable gait speed), fatigue (FACIT-F), pain, walking difficulties Walk-12G, fear of falling (FES[S]), history of falls, history of near falls, balance problems while dual tasking, need help from others in daily activities (PADLS), freezing (FOGQsa, item 3), turning hesitations (FOGQsa, item 6), retropulsion (NRT).

^3^ Possible score range, 0–130; for the regression analysis, scores were adjusted so that higher scores = more problems.

BBS, Berg Balance Scale; FACIT-F, the Functional Assessment of Chronic Illness Therapy; FES(S), Falls Efficacy Scale, Swedish version; FOGQsa, Freezing of Gait Questionnaire, self-administered version; MMSE, Mini Mental State Examination; NRT, Nutt Retropulsion Test; PADLS, the Parkinson’s disease Activities of Daily Living Scale; PD, Parkinson’s disease; UPDRS III, part III (motor score) of the Unified PD Rating Scale; 10MWT, 10-Meter Walk Test, m/s, meters per second; Walk-12G, 12-item generic walking scale.

### Predictors of prospective falls

Simple logistic regression analyses identified 15 independent variables associated with prospective falls at P<0.2; the identified variables were the same as in Table 3 except for MMSE (P = 0.73). Four independent predictors for prospective falls were identified (OR, 95% CI): pain (4.9, 1.8–13.5), history of near falls (3.3, 1.3–8.3), retropulsion (3.5, 1.3–9.4) and disease duration (1.2, 1.0–1.3). Details from these analyses are available on request.

## Discussion

This study comprehensively investigated contributing factors for experiencing future falls and/or near falls in people mildly affected by PD. When using multivariate analyses, three contributing factors were identified. The strongest factor was FOF, followed by a history of near falls and having retropulsion. That is, FOF seems to be an important issue to address already in mild PD as well as asking about prior near falls. Our findings may thus have important clinical implications since these aspects may not be addressed in those having mild PD. Although several prospective studies have investigated contributing factors for experiencing future falls (e.g, [17–20,41–44]) few included near falls as an independent or dependent variable [20]. This study thus contributes to the body of knowledge since it is imperative to early on detect those at risk in order to work proactively.

To the best of our knowledge, this is the first study presenting FOF as an independent associated factor for experiencing future falls and/or near falls in people with mild PD, although it has been identified as an independent risk factor for recurrent falls [11]. FOF in people with PD is also of importance since it is a major barrier to physical exercise [45]; it may cause activity avoidance, participation restrictions and social isolation [44,46,47] and is negatively associated with health-related quality of life [48]. Taken together, FOF should probably be considered an integrate part of PD-assessments irrespective of disease severity.

The second strongest independent factor was a history of near falls. Although it has been suggested that one should ask people with PD about prior near falls [12] a study by Ashburn et al. did not support that near falls during the past year predicted future falls [20]. In that study, near falls was defined as “occasions on which individuals felt that they were going to fall but did not actually do so” [12]. Besides using a different definition, future falls were collected based on retrospective recall covering a shorter period (3 months) than the prospective 6-month follow-up used here. Our finding indicates that asking about prior near falls as defined by Gray et al. [39] may be helpful in identifying persons with mild PD that are at risk for future falls and/or near falls. Furthermore, it needs to be noted that during the 6-month follow-up, the proportion of near falls incidences far outweighed that for falls (65% versus 35%).

We suggest that near falls deserve more attention in PD research to gain an increased knowledge about associated factors, consequences and whether near falls is a precursor of falls. The latter requires longitudinal studies. There might also be a need for studies of how to best monitor and register near falls incidences.

In contrast to near falls, a history of falls was not identified as a risk factor for future falls and/or near falls. This finding is in contrast to several previous studies (e.g. [18–20,42,44]. The discrepancy might be due to that our dependent variable included both near falls and/or falls, and that a history of near falls was included as an independent variable, which has not been the case in previous studies. Another explanation might be that our sample represented relatively mild PD. For example, the proportion of participants that prospectively reported falls (32%) is lower compared to other prospective studies of falls in PD (range, 35–90%) [11]. However, in another study that investigated falls prospectively in people with mild PD about 68% reported fall [19]. Methodological aspects may also play a part in the number of falls reported. In the study by Wood et al., each subject was given a set of weekly prepaid postcards to return for one year. A fall report was followed up by telephone to outline the exact circumstances of the fall event. If cards were not returned one week after their expected return date, this would also prompt telephone contact [19].

Still, a history of near falls but not falls was identified as a risk factor when excluding near falls from the dependent variable. This may indicate that near falls is a precursor of experiencing future falls [22,23], suggesting that it may be more effective to ask about prior near falls than actual falls if you aim at working pro-actively. Additional studies are needed to support or refute the present findings and to understand the relationships between near falls and falls.

The third independent associated factor identified was retropulsion according to the NRT, which was positive in 25% of our participants. In relatively mild PD, this might be seen as a surprising finding. However, postural instability has been shown to be present already at diagnosis [6] although it worsens with disease progression. The Sydney multicenter longitudinal study reported that 34% demonstrated postural instability two years after diagnosis [7] which increased to 71% after ten years [49]. In the present study, the reasoning for choosing the NRT as a pull test is that it incorporates an unexpected shoulder pull and only one trial is performed; this version of the pull test has been suggested to provide a more valid evaluation that reflects everyday life situations [4].

Some methodological limitations and considerations need to be acknowledged. This study involves people with mild PD but people being above the age of 80 years were not included. Our findings may therefore not be applicable to very old people with mild PD. Although several independent variables were included, several other variables may contribute to the occurrence of falls and near falls. Furthermore, some of the included variables that were not shown to be independently associated with prospective falls/near falls were assessed by using relative rough indicators. For instance, to capture those having mild cognitive impairments in PD, the Montreal Cognitive Assessment (MoCA) has been suggested to be preferably to MMSE [50,51]. In addition, several variables (e.g. dyskinesia, freezing of gait and turning hesitations) were dichotomized, which may lead to loss of information. However, this was done for reasons related to the distributional properties of item responses. We also acknowledge that retrospective recall of near falls may be more problematic than for falls. In this study, no retrospective time frame was used and whether this influenced the results is unclear. Future studies are needed to address the potential impact of using a retrospective time frame (e.g. six or twelve months) in relation to history of near falls in people with PD.

## Conclusions

This study identified three contributing factors for experiencing future falls and/or near falls in people mildly affected by their PD. The strongest factor was FOF, followed by a history of near falls and having retropulsion. That is, FOF seems to be an important issue to address already in mild PD as well as asking about prior near falls. A history of near falls appears to be a stronger predictor for future falls than a history of falls. This highlights the need for addressing near falls in more depth in larger longitudinal studies including a broader range of PD severities.

## References

[1] Nieuwboer A, De Weerdt W, Dom R, Lesaffre E (1998) A frequency and correlation analysis of motor deficits in Parkinson patients. Disabil Rehabil 20: 142–150. 9571381 10.3109/09638289809166074

[2] Carpenter MG, Allum JH, Honegger F, Adkin AL, Bloem BR (2004) Postural abnormalities to multidirectional stance perturbations in Parkinson’s disease. J Neurol Neurosurg Psychiatry 75: 1245–1254. 15314109 10.1136/jnnp.2003.021147 PMC1739252

[3] Horak FB, Dimitrova D, Nutt JG (2005) Direction-specific postural instability in subjects with Parkinson’s disease. Exp Neurol 193: 504–521. 15869953 10.1016/j.expneurol.2004.12.008

[4] Visser M, Marinus J, Bloem BR, Kisjes H, van den Berg BM, et al (2003) Clinical tests for the evaluation of postural instability in patients with parkinson’s disease. Arch Phys Med Rehabil 84: 1669–1674. 14639568 10.1053/s0003-9993(03)00348-4

[5] Berg KO, Wood-Dauphinee SL, Williams JI, Maki B (1992) Measuring balance in the elderly: validation of an instrument. Can J Public Health 83 Suppl 2: S7–11. 1468055

[6] Hariz GM, Forsgren L (2011) Activities of daily living and quality of life in persons with newly diagnosed Parkinson’s disease according to subtype of disease, and in comparison to healthy controls. Acta Neurol Scand 123: 20–27. 10.1111/j.1600-0404.2010.01344.x 20199514

[7] Hely MA, Morris JG, Rail D, Reid WG, O’Sullivan DJ, et al (1989) The Sydney Multicentre Study of Parkinson’s disease: a report on the first 3 years. J Neurol Neurosurg Psychiatry 52: 324–328. 2647907 10.1136/jnnp.52.3.324 PMC1032404

[8] Kelly VE, Eusterbrock AJ, Shumway-Cook A (2012) A review of dual-task walking deficits in people with Parkinson’s disease: motor and cognitive contributions, mechanisms, and clinical implications. Parkinsons Dis 2012: 918719 10.1155/2012/918719 22135764 PMC3205740

[9] Nilsson MH, Hariz GM, Iwarsson S, Hagell P (2012) Walking ability is a major contributor to fear of falling in people with Parkinson’s disease: implications for rehabilitation. Parkinsons Dis 2012: 7. 10.1155/2012/713236 PMC3175698 21941686

[10] Lindholm B, Hagell P, Hansson O, Nilsson MH (2014) Factors associated with fear of falling in people with Parkinson’s disease. BMC Neurol 14: 19 10.1186/1471-2377-14-19 24456482 PMC3904169

[11] Allen NE, Schwarzel AK, Canning CG (2013) Recurrent falls in Parkinson’s disease: a systematic review. Parkinsons Dis 2013: 906274 10.1155/2013/906274 23533953 PMC3606768

[12] Stack E, Ashburn A (1999) Fall events described by people with Parkinson’s disease: implications for clinical interviewing and the research agenda. Physiother Res Int 4: 190–200. 10581625 10.1002/pri.165

[13] Ashburn A, Stack E, Pickering RM, Ward CD (2001) A community-dwelling sample of people with Parkinson’s disease: characteristics of fallers and non-fallers. Age Ageing 30: 47–52. 11322672 10.1093/ageing/30.1.47

[14] Ashburn A, Fazakarley L, Ballinger C, Pickering R, McLellan LD, et al (2007) A randomised controlled trial of a home based exercise programme to reduce the risk of falling among people with Parkinson’s disease. J Neurol Neurosurg Psychiatry 78: 678–684. 17119004 10.1136/jnnp.2006.099333 PMC2117667

[15] Nilsson MH, Rehncrona S, Jarnlo GB (2011) Fear of falling and falls in people with Parkinson’s disease treated with deep brain stimulation in the subthalamic nuclei. Acta Neurol Scand 123: 424–429. 10.1111/j.1600-0404.2010.01418.x 21492098

[16] Jonasson SB, Nilsson MH, Lexell J (2014) Psychometric properties of four fear of falling rating scales in people with Parkinson’s disease. BMC Geriatr 14: 66 10.1186/1471-2318-14-66 24884466 PMC4035736

[17] Kerr GK, Worringham CJ, Cole MH, Lacherez PF, Wood JM, et al (2010) Predictors of future falls in Parkinson disease. Neurology 75: 116–124. 10.1212/WNL.0b013e3181e7b688 20574039

[18] Latt MD, Lord SR, Morris JG, Fung VS (2009) Clinical and physiological assessments for elucidating falls risk in Parkinson’s disease. Mov Disord 24: 1280–1289. 10.1002/mds.22561 19425059

[19] Wood BH, Bilclough JA, Bowron A, Walker RW (2002) Incidence and prediction of falls in Parkinson’s disease: a prospective multidisciplinary study. J Neurol Neurosurg Psychiatry 72: 721–725. 12023412 10.1136/jnnp.72.6.721 PMC1737913

[20] Ashburn A, Stack E, Pickering RM, Ward CD (2001) Predicting fallers in a community-based sample of people with Parkinson’s disease. Gerontology 47: 277–281. 11490147 10.1159/000052812

[21] Lamb SE, Jorstad-Stein EC, Hauer K, Becker C (2005) Development of a common outcome data set for fall injury prevention trials: the Prevention of Falls Network Europe consensus. J Am Geriatr Soc 53: 1618–1622. 16137297 10.1111/j.1532-5415.2005.53455.x

[22] Teno J, Kiel DP, Mor V (1990) Multiple stumbles: a risk factor for falls in community-dwelling elderly. A prospective study. J Am Geriatr Soc 38: 1321–1325. 2254571 10.1111/j.1532-5415.1990.tb03455.x

[23] Sipp AR, Rowley BA (2008) Detection of baseline and near-fall postural stability. Conf Proc IEEE Eng Med Biol Soc 2008: 1262–1265. 10.1109/IEMBS.2008.4649393 19162896

[24] Giladi N, Shabtai H, Simon ES, Biran S, Tal J, et al (2000) Construction of freezing of gait questionnaire for patients with Parkinsonism. Parkinsonism Relat Disord 6: 165–170. 10817956 10.1016/s1353-8020(99)00062-0

[25] Giladi N, Tal J, Azulay T, Rascol O, Brooks DJ, et al (2009) Validation of the freezing of gait questionnaire in patients with Parkinson’s disease. Mov Disord 24: 655–661. 10.1002/mds.21745 19127595

[26] Nilsson MH, Hariz GM, Wictorin K, Miller M, Forsgren L, et al (2010) Development and testing of a self administered version of the Freezing of Gait Questionnaire. BMC Neurol 10: 85 10.1186/1471-2377-10-85 20863392 PMC2955563

[27] Bladh S, Nilsson MH, Hariz GM, Westergren A, Hobart J, et al (2012) Psychometric performance of a generic walking scale (Walk-12G) in multiple sclerosis and Parkinson’s disease. J Neurol 259: 729–738. 10.1007/s00415-011-6254-z 21956376

[28] Yellen SB, Cella DF, Webster K, Blendowski C, Kaplan E (1997) Measuring fatigue and other anemia-related symptoms with the Functional Assessment of Cancer Therapy (FACT) measurement system. J Pain Symptom Manage 13: 63–74. 9095563 10.1016/s0885-3924(96)00274-6

[29] Hagell P, Hoglund A, Reimer J, Eriksson B, Knutsson I, et al (2006) Measuring fatigue in Parkinson’s disease: a psychometric study of two brief generic fatigue questionnaires. J Pain Symptom Manage 32: 420–432. 17085268 10.1016/j.jpainsymman.2006.05.021

[30] Tinetti ME, Richman D, Powell L (1990) Falls efficacy as a measure of fear of falling. J Gerontol 45: 239–243. 10.1093/geronj/45.6.p239 2229948

[31] Nilsson MH, Drake AM, Hagell P (2010) Assessment of fall-related self-efficacy and activity avoidance in people with Parkinson’s disease. BMC Geriatr 10: 78 10.1186/1471-2318-10-78 20973974 PMC2984450

[32] Hobson JP, Edwards NI, Meara RJ (2001) The Parkinson’s Disease Activities of Daily Living Scale: a new simple and brief subjective measure of disability in Parkinson’s disease. Clin Rehabil 15: 241–246. 11386393 10.1191/026921501666767060

[33] Hagell P, Hariz GM, Nilsson MH (2009) The Parkinson’s disease Activities of Daily Living Scale (PADLS) revisited. Parkinsonism Relat Disord 15(Suppl 2): S62.

[34] Steffen T, Seney M (2008) Test-retest reliability and minimal detectable change on balance and ambulation tests, the 36-item short-form health survey, and the unified Parkinson disease rating scale in people with parkinsonism. Phys Ther 88: 733–746. 10.2522/ptj.20070214 18356292

[35] Nutt J, Hammerstad J, Gancher S (1992) Diagnosis:Is it Parkinsonism?-Major symptoms and signs of the disorder Parkinson’s disease: 100 maxims. London: Edward Arnold pp. 3–9. 10.1111/jvh.12392

[36] Fahn S, Elton R, et.al (1987) Unified Parkinson’s Disease Rating Scale In: Fahn S, Marsden CD, Calne D, Goldstein M, editors. Recent developments in Parkinson’s disease. Florham Park, NJ:: McMillan Healthcare Information pp. 153–163, 293–304.

[37] Folstein MF, Folstein SE, McHugh PR (1975) "Mini-mental state". A practical method for grading the cognitive state of patients for the clinician. J Psychiatr Res 12: 189–198. 1202204 10.1016/0022-3956(75)90026-6

[38] Hoehn MM, Yahr MD (2001) Parkinsonism: onset, progression, and mortality. 1967. Neurology 57: S11–26. 11775596

[39] Gray P, Hildebrand K (2000) Fall risk factors in Parkinson’s disease. J Neurosci Nurs 32: 222–228. 10994536 10.1097/01376517-200008000-00006

[40] Tomlinson CL, Stowe R, Patel S, Rick C, Gray R, et al (2010) Systematic review of levodopa dose equivalency reporting in Parkinson’s disease. Mov Disord 25: 2649–2653. 10.1002/mds.23429 21069833

[41] Allan LM, Ballard CG, Rowan EN, Kenny RA (2009) Incidence and prediction of falls in dementia: a prospective study in older people. PLoS One 4: e5521 10.1371/journal.pone.0005521 19436724 PMC2677107

[42] Allcock LM, Rowan EN, Steen IN, Wesnes K, Kenny RA, et al (2009) Impaired attention predicts falling in Parkinson’s disease. Parkinsonism Relat Disord 15: 110–115. 10.1016/j.parkreldis.2008.03.010 18487069

[43] Cole MH, Silburn PA, Wood JM, Worringham CJ, Kerr GK (2010) Falls in Parkinson’s disease: kinematic evidence for impaired head and trunk control. Mov Disord 25: 2369–2378. 10.1002/mds.23292 20737542

[44] Bloem BR, Grimbergen YA, Cramer M, Willemsen M, Zwinderman AH (2001) Prospective assessment of falls in Parkinson’s disease. J Neurol 248: 950–958. 11757958 10.1007/s004150170047

[45] Ellis T, Boudreau JK, Deangelis TR, Brown LE, Cavanaugh JT, et al (2013) Barriers to exercise in people with Parkinson disease. Phys Ther 93: 628–636. 10.2522/ptj.20120279 23288910 PMC3641403

[46] Brozova H, Stochl J, Roth J, Ruzicka E (2009) Fear of falling has greater influence than other aspects of gait disorders on quality of life in patients with Parkinson’s disease. Neuro Endocrinol Lett 30: 453–457. 20010494

[47] Thordardottir B, Nilsson MH, Iwarsson S, Haak M (2014) "You plan, but you never know"—participation among people with different levels of severity of Parkinson’s disease. Disabil Rehabil. 24670191 10.3109/09638288.2014.898807

[48] Grimbergen YA, Schrag A, Mazibrada G, Borm GF, Bloem BR (2013) Impact of falls and fear of falling on health-related quality of life in patients with Parkinson’s disease. J Parkinsons Dis 3: 409–413. 10.3233/JPD-120113 23948987

[49] Hely MA, Morris JG, Traficante R, Reid WG, O’Sullivan DJ, et al (1999) The sydney multicentre study of Parkinson’s disease: progression and mortality at 10 years. J Neurol Neurosurg Psychiatry 67: 300–307. 10449550 10.1136/jnnp.67.3.300 PMC1736543

[50] Lessig S, Nie D, Xu R, Corey-Bloom J (2012) Changes on brief cognitive instruments over time in Parkinson’s disease. Mov Disord 27: 1125–1128. 10.1002/mds.25070 22692724

[51] Dalrymple-Alford JC, MacAskill MR, Nakas CT, Livingston L, Graham C, et al (2010) The MoCA: well-suited screen for cognitive impairment in Parkinson disease. Neurology 75: 1717–1725. 10.1212/WNL.0b013e3181fc29c9 21060094

